# Remineralization potential of fluoridated and non-fluoridated toothpastes on induced dental caries: A comparative *in vitro* analysis

**DOI:** 10.6026/973206300212035

**Published:** 2025-07-31

**Authors:** Ashjan Ashraf Batha, Udita Samanta, Sujata Datta, Apurva Chadha, Shipra Jaidka, Deepti Jawa

**Affiliations:** 1 Department of Paediatric and Preventive Dentistry, Divya Jyoti College of Dental Science and Research, Modinagar, Uttar Pradesh 201204, India

**Keywords:** Dental caries, enamel demineralization, fluoridated toothpaste, microhardness

## Abstract

Dental caries is a global concern marked by enamel demineralization due to bacterial activity. This *in vitro* study compared the remineralization efficacy of fluoridated and non-fluoridated dentifrices. Fifty demineralized premolars were divided into five groups and treated for 30 days. Nano hydroxyapatite demonstrated the highest surface microhardness improvement, followed by sodium fluoride, calcium sucrose phosphate and EnamelMax. All tested agents showed remineralizing potential, with nano-hydroxyapatite being the most effective.

## Background:

Dental caries is one of the oldest and most prevalent chronic infectious diseases, with historical records dating back to 5000 BC, when it was attributed to a "tooth worm." The term "dental caries," originating from the Latin word caries, meaning decay, first appeared in literature around 1634. Despite advancements in preventive measures such as fluoride application, dental sealants and improved oral hygiene practices, dental caries continues to affect nearly 100% of adults worldwide, with its global prevalence remaining relatively stable over the past three decades. This persistence can be attributed to its multifactorial etiology, involving microbial, dietary, genetic and socioeconomic factors [1]. Caries develops when bacteria on the tooth surface metabolize dietary sugars into acids, which demineralize the enamel. According to Miller's Chemoparasitic Theory, acids produced from the fermentation of carbohydrates are responsible for dissolving tooth minerals [2]. The caries process is dynamic, characterized by alternating phases of demineralization and remineralization, the latter occurring when calcium and phosphate ions are present to help rebuild enamel [3]. The disease is primarily initiated by Streptococcus mutans and Lactobacilli, which produce acid through carbohydrate fermentation. Newbrun later expanded Keyes' Triad by identifying time as an additional critical factor in the development of caries. Preventive strategies thus emphasize reducing sugar intake, maintaining oral hygiene and using remineralizing agents that enhance enamel resistance and arrest early carious lesions [4]. Professional interventions such as fluoride treatments, sealants and cleanings are important but may be costly or inaccessible. Therefore, daily at-home care-including the use of remineralizing dentifrices-is essential. Fluoridated agents, known to enhance enamel resistance, require calcium and phosphate from saliva and pose fluorosis risks when over consumed, particularly in children [5, 6]. Non-fluoridated remineralizing agents are gaining attention due to their lower toxicity and independent ion-supplying mechanisms. These include calcium sucrose phosphate, calcium sodium phosphosilicate, nano-hydroxyapatite and casein phosphopeptide-amorphous calcium phosphate, among others. These agents support subsurface enamel remineralization by releasing or stabilizing essential ions and functioning even at acidic pH. Additional ingredients like arginine, xylitol and sodium trimetaphosphate further enhance enamel repair and reduce plaque formation [7]. Therefore, it is of interest to evaluate and compare the remineralizing potential of selected fluoridated and non-fluoridated dentifrices-specifically Nano hydroxyapatite, Calcium sucrose phosphate, EnamelMax (containing xylitol, arginine and sodium trimetaphosphate) and Sodium fluoride-on artificially induced caries lesions using the Vickers hardness test in an *in vitro* setting.

## Materials and Methods:

The study involved 50 freshly extracted human maxillary and mandibular bicuspids, which were divided into five groups, each containing 10 teeth. Group A served as the control group, where no remineralization agent was applied, while the experimental groups (B, C, D, E) were treated with different dentifrices containing Nano-hydroxyapatite (Group B), Calcium sucrose phosphate (Group C), EnamelMax (xylitol + arginine + sodium trimetaphosphate) (Group D) and Sodium fluoride (Group E). The sample selection was based on strict inclusion and exclusion criteria, ensuring that the selected teeth were free from caries, restorations, or fluorosis. Each tooth underwent a series of preparatory steps, including ultrasonic scaling, sectioning to retain only the buccal halves and embedding in acrylic moulds to standardize the surface for remineralization treatment. The teeth were then coated with acid-resistant nail varnish, leaving a 2mm x 4mm window for treatment. To simulate early stages of tooth decay, samples were immersed in demineralizing solution for 96 hours and surface micro hardness was measured using a Vickers Hardness Test before and after demineralization. Following demineralization, each group of samples received the assigned treatment twice daily for two minutes over one month, which were stored in artificial saliva to simulate oral conditions, 60 ml of artificial saliva measured by a measuring cup was used for each group which was renewed every 24 h just before immersion of freshly treated samples [8]. The dentifrices used in the experimental groups contained respective remineralization agents and the surface hardness was re-evaluated to assess the effectiveness of each agent in remineralizing the enamel. Throughout the study, strict ethical guidelines were followed and the procedure was conducted under regulated conditions.

## Ethical considerations:

The study was approved by the ethical committee under the clearance number DJC/IEC/29/2025.

## Data collection and analysis:

Data was collected using digital Vickers hardness testing at three stages: baseline, post-demineralization and post-remineralization. The values were recorded using ToupView software and processed in Microsoft Excel. Statistical analysis was performed using SPSS Version 23.0, employing One-Way ANOVA for intergroup comparisons and paired t-tests/Wilcoxon signed-rank tests for intragroup comparisons. The significance level was set at 5%, with normality and homogeneity assessed using the Shapiro-Wilk and Levene's tests, respectively. This methodological framework ensures reliability and validity in evaluating the effectiveness of different remineralizing agents on enamel lesions.

## Results:

All groups showed statistically significant reductions in surface microhardness after demineralization (p = 0.001). Group B (Nano hydroxyapatite) showed the highest percentage reduction (49.79%), followed closely by Group E (Sodium fluoride) and Group D (EnamelMax). The lowest reduction was in Group A (Control), suggesting uniform demineralization across samples. One-way ANOVA confirmed significant differences among the groups (F = 69.240, p = 0.001) (Table 1 - see PDF). Post-remineralization, all experimental groups demonstrated significant improvements in surface hardness. Group B (Nano hydroxyapatite) showed the highest remineralization (75.71%), followed by Group E (Sodium fluoride) and Group C (Calcium sucrose phosphate). Group A (Control) showed minimal change, confirming no spontaneous remineralization without treatment. One-way ANOVA confirmed intergroup significance (F = 69.240, p = 0.001) (Table 2 - see PDF). Post-hoc analysis using Tukey's HSD revealed significant differences between all pairs (p = 0.001). Nano hydroxyapatite (Group B) had significantly greater remineralization than all other agents, highlighting its superior efficacy. Control (Group A) showed the least effect, validating the active role of the tested remineralizing agents (Table 3 - see PDF).

## Discussion:

According to the World Health Organization (2018), dental caries is the most prevalent chronic disease globally, affecting 60% to 90% of school-aged children and a vast majority of adults. Dental caries is a sugar-driven, biofilm-mediated disease that disrupts the balance between demineralization and remineralization, ultimately leading to enamel loss and cavity formation [9]. The development of caries is influenced by both protective and pathological factors. Protective factors include saliva, which provides buffering capacity, cleansing effects and antibacterial properties, while external aids such as fluoride, sealants and proper oral hygiene help in caries prevention [10]. On the other hand, pathogenic factors include dietary habits, bacterial activity and plaque formation [11]. When the balance tips in favor of demineralization, the enamel undergoes mineral loss, leading to white spot lesions-early indicators of caries [12]. Preventing demineralization necessitates neutralizing oral pH and increasing remineralization, which can be accomplished naturally through calcium and phosphate ions in saliva or artificially with remineralizing agents. Fluoride has been the cornerstone of remineralization efforts for over a century. Dr. Frederick McKay first observed fluoride-induced dental staining in 1901, which later led to the discovery of fluoride's role in caries prevention. Dean's extensive research in the 1930s and 1940s confirmed that 1 ppm fluoride in drinking water could reduce caries incidence by 60% [13]. Fluoride functions by promoting remineralization through the formation of fluorapatite, which enhances enamel resistance to acid attacks [14]. The effectiveness of fluoride-containing toothpaste, which typically has sodium monofluorophosphate or conventional sodium fluoride, is well established [15]. However, excessive fluoride intake can lead to dental fluorosis and potential toxicity [16]. Due to concerns about fluoride toxicity, researchers have explored alternative remineralizing agents. Non-fluoridated remineralization systems work independently of saliva quality and do not require strict application regimens. They offer effective remineralization for pit and fissure as well as smooth surface caries. With newer non-fluoridated techniques, we can re-establish the health of oral tissues without the adverse effects of fluorides. Nano-Hydroxyapatite (nHAp) is particularly promising as it closely mimics the natural mineral composition of enamel. Due to its nanoscale size, it can integrate into demineralized areas, filling porosities and serving as a scaffold for calcium and phosphate attraction [17].

Calcium sucrose phosphate, also known as anticay, is a vital remineralizing agent for strengthening teeth and preventing decay. It consists of calcium, sucrose and phosphate, with 11.5% calcium by dry mass. It reduces enamel solubility in acidic environments. Xylitol enhances salivary flow rate, prevents demineralization and increases plaque pH, buffering capacity and remineralization by increasing calcium, phosphate and hydroxyl ions concentration in saliva. Trimetaphosphate ion (TMP) in sodium trimetaphosphate (STMP) adsorbs to the enamel surface, creating a barrier coating that reduces demineralization during acid challenge. Arginine, an amino acid found in saliva, is metabolized by oral microbial flora into ornithine, ammonia and carbon dioxide, inhibiting demineralization and playing a crucial role in mineralizing hydroxyapatite. Recently, EnamelMax, the combination of xylitol, arginine and sodium trimetaphosphate in oral health, primarily enhances enamel remineralization, reduces demineralization and balances the oral microbiome. The need for non-fluoridated remineralization agents in toothpastes is growing due to the growing demand for fluoride-free toothpaste. The study used 50 human permanent premolar teeth. Premolars were selected due to their uniform enamel composition, reducing variability in demineralization and remineralization assessments [18]. The Vickers hardness test was employed for microhardness evaluation, as it is a widely standardized, non-destructive and reliable method [19]. The study involved the creation of artificial carious lesions through exposure to a demineralizing solution at pH 4.5 for 96 hours at 37°C, a scientifically validated method for simulating early enamel caries [20]. The samples were then stored in artificial saliva to mimic the remineralizing role of natural saliva, ensuring consistent mineral availability (Amaechi *et al.* 1999) [8]. Brushing regimens were standardized to two minutes twice daily for 30 days, by recommendations from the American Dental Association (ADA) and World Health Organization (WHO) [21]. Among the tested agents, nano-hydroxyapatite exhibited the highest remineralizing potential, followed by sodium fluoride, calcium sucrose phosphate and EnamelMax (a combination of xylitol, arginine and STMP). The superior performance of nano-hydroxyapatite is attributed to its bioactivity, biocompatibility and ability to integrate directly into the enamel matrix, enhancing mineral density restoration. Recent studies have shown that nano-hydroxyapatite has superior remineralization potential compared to conventional fluoride-based agents. Sodium fluoride was also effective, but it relies on the availability of calcium and phosphate ions in the oral environment for optimal performance. In a study by Gayan *et al.* in 2025, sodium fluoride and casein phosphopeptide-amorphous calcium phosphate were the most effective remineralizing agents for enhancing the surface microhardness of artificially demineralized enamel in primary molars, with sodium fluoride exhibiting the highest surface microhardness among all the groups [22]. Nano-hydroxyapatite showed moderate efficacy, whereas GSE and normal saline exhibited limited or no significant remineralizing potential. The superior performance of sodium fluoride and CPP-ACP highlights their potential as primary therapeutic options for managing early enamel lesions in pediatric dentistry. Calcium sucrose phosphate exhibited moderate efficacy, while Enamel Max produced a synergistic yet less pronounced remineralizing effect. As far as we know, this is the first study to evaluate the remineralizing potential of this synergistic combination. Notably, slight remineralization was observed even in the control group, likely due to the calcium and phosphate present in artificial saliva [23]. In a similar study by Matkar *et al.* in 2023, buccal surface of eighty extracted human premolars were treated with demineralization solution and randomly distributed into four groups (control, Xyliwhite toothpaste (fluoride free), Superbee Propolis toothpaste (fluoride free), and Amflor Pro toothpaste (Fluoridated). Results showed that all 3 other experimental groups had higher remineralization efficacy as compared to control (P < 0.001), but maximum remineralization efficacy was seen with Fluoridated Toothpaste (Amflor Pro) [24]. Moreover, tooth brushing, regardless of the use of a remineralizing agent, may have caused micro abrasions that exposed enamel surfaces to minerals in the saliva, facilitating mild remineralization. These findings support the potential use of non-fluoridated agents as alternatives to fluoride in promoting enamel repair, while also addressing concerns about fluoride toxicity. To the best of our knowledge, this is the first *in vitro* study comparing the remineralization potential of these four remineralizing agents altogether. However, results obtained *in vitro* may be quite different because of their obvious limitations compared to the dynamic complex biological system, which usually occurs in the oral cavity *in vivo*. Therefore, long-term clinical trials and further research need to be done in order to establish the efficacy and superiority of these test agents.

## Conclusion:

The growing potential of non-fluoridated remineralization agents as effective options for caries prevention and enamel repair is shown. Among these, nano-hydroxyapatite is a promising alternative due to its ability to integrate with the enamel structure and support ongoing mineral restoration.
